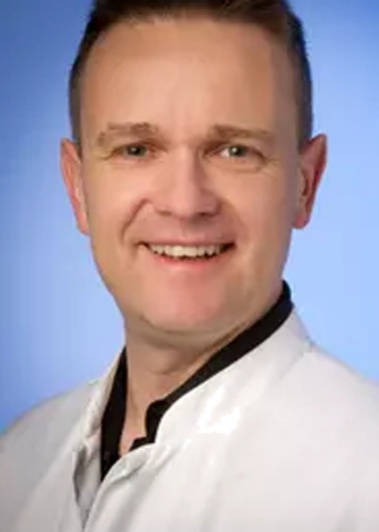# Principles and Applications of Miniaturized Near‐Infrared (NIR) Spectrometers

**DOI:** 10.1002/chem.202002838

**Published:** 2020-10-29

**Authors:** Krzysztof B. Beć, Justyna Grabska, Christian W. Huck

**Affiliations:** ^1^ Institute of Analytical Chemistry and Radiochemistry CCB-Center for Chemistry and Biomedicine Leopold-Franzens University Innrain 80/82 6020 Innsbruck Austria

**Keywords:** analytical methods, handheld devices, IR spectroscopy, miniaturized spectrometers, near-infrared spectroscopy

## Abstract

This review article focuses on the principles and applications of miniaturized near‐infrared (NIR) spectrometers. This technology and its applicability has advanced considerably over the last few years and revolutionized several fields of application. What is particularly remarkable is that the applications have a distinctly diverse nature, ranging from agriculture and the food sector, through to materials science, industry and environmental studies. Unlike a rather uniform design of a mature benchtop FTNIR spectrometer, miniaturized instruments employ diverse technological solutions, which have an impact on their operational characteristics. Continuous progress leads to new instruments appearing on the market. The current focus in analytical NIR spectroscopy is on the evaluation of the devices and associated methods, and to systematic characterization of their performance profiles.

## Graphical abstract

**Small scale**: Miniaturized spectrometers have revolutionized near‐infrared spectroscopy by opening up a spectrum of new applications for this already mature analytical technique. This review presents current issues in this thriving field. The diversity of underlying technology, miniaturization versus performance, and very active research areas of systematic feasibility studies aimed at establishing the applicability of these devices are highlighted.

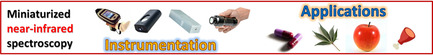

## 1 Introduction

Near‐infrared (NIR) spectroscopy has gained remarkable value as a nondestructive analytical technique and it has become the tool of choice in several fields of application.[^1^, ^2^] Its primary advantages in practical roles, 1) applicability to a wide variety of samples; and 2) rapid, noninvasive analysis, form a good synergy with the autonomous, portable spectrometers that are capable of on‐site analysis. Such NIR spectrometers have emerged in the past decade and led to a significant leap in the evolution of the practical applications of this technique.[^3^, ^4^] Nevertheless, several issues connected with the peculiarity of miniaturized spectrometers have become apparent. In contrast to the mature design of a FTNIR benchtop spectrometer, handheld devices are much less uniform and implement diverse and novel technological solutions. This results in differing performance profiles of miniaturized spectrometers from that of laboratory instruments and between each model as well. The most apparent distinctiveness is the narrower spectral regions and/or lower spectral resolution with which the compact devices operate. For these reasons, current research focus is directed to a thorough systematic evaluation of the applicability limits and analytical performance of such devices in a variety of applications. The scope of this review is to provide comprehensive information on miniaturized NIR spectrometers, including the principles of the technology, current applications, and the potential for future advances.

### 1.1 Practical importance and remaining challenges in the application of miniaturized NIR spectroscopy

The value of NIR spectroscopy in analytical chemistry results from combined physicochemical and instrumental reasons. These are briefly discussed in Sections 2.1 and 2.2. Here, attention should be given to the primary driver behind the adoption of NIR spectroscopy in a variety of practical roles. Often, it is a feasible alternative to time‐inefficient and resource‐intensive conventional methods of analysis, such as HPLC. Within the established framework of NIR spectral analysis, these demanding methods are required only once, and their role is to provide reference data for subsequent calibration. Once a reliable calibration model that links the measured NIR spectra with a given property of the sample (e.g., concentration of a selected compound or a group of compounds available from reference analysis) is established, rapid and efficient spectral measurements can substitute the less efficient analytical method in further routines.^[5]^ Consequently, the greatest gain from the application of NIR spectroscopy is in the analyses, in which a large amount of samples of relatively uniform properties are used. NIR spectroscopy is therefore widely adopted in high‐throughput analysis in agriculture and various industries. In this scheme of an efficient, short‐time‐to‐result method, the bottleneck limitation originally was the still unavoidable laboratory setup for spectral measurements. Therefore, the appearance of autonomous, portable NIR spectrometers could be seen as a major breakthrough in several established roles. Furthermore, this leap offered the development of entirely new, previously unattainable applications. Scenarios in which spectral measurements are necessary directly on‐site became possible, a factor that meets keen interest from, for example, agri‐food or natural medicine industry. The potential of portable NIR spectroscopy in such applications has repeatedly been demonstrated.

Notably, the miniaturization of the instrumentation is not solely specific to NIR spectroscopy. Rather, it is a trend observed throughout widely understood spectroscopy and spectrometry.^[3]^ In certain applications, attenuated total reflection infrared (ATR‐IR) or Raman techniques are possible alternatives to NIR spectroscopy.[^6^, ^7^] In both cases, the portable devices cannot match neither the affordability nor the compact factor of miniaturized NIR spectrometers. On the other hand, certain other techniques for which instruments with such features are available, for example, fluorescence, are inferior to NIR spectroscopy in fundamental capabilities, such as the chemical specificity of the method and applicability to a wide selection of samples.^[4]^

The number of reports in the literature on miniaturized NIR spectrometers is rapidly increasing nowadays, reflecting the sharp edge in applicability of this technology to conventional spectroscopy that is limited to laboratory use. However, the revolutionary step into miniaturization has required implementing new technological solutions, which unequivocally affected the performance of the portable NIR spectrometers. Several distinct design principles have been implemented in the instruments introduced into the market over the past decade. Furthermore, in several applications, the cost‐per‐unit of portable spectrometers is critical for wide adoption, and there is an economic stimulus for offering highly affordable instruments. Given these two reasons, operating characteristics largely differ between the available miniaturized NIR spectrometers. Systematic feasibility studies are necessary to evaluate the accuracy and robustness, in an analytical sense, of these instruments in various applications. Currently, this is an active area of research in analytical chemistry.

## 2 Essential Background

Unique strengths and limitations of miniaturized NIR spectroscopy result from the underlying factors of both physical and instrumental nature. Prior to discussing these features, necessary key information is provided, and the interested reader will find more exhaustive information in the referenced literature.

### 2.1 Physical principles of NIR spectroscopy

NIR spectroscopy extracts information from the sample through molecular vibrational excitations, similar to IR and Raman techniques. However, the principle difference between NIR spectroscopy and the last two techniques is that, in the NIR spectral region (typically defined as 12 500–4000 cm^−1^ or 800–2500 nm), only overtones and combination transitions can be observed.

These are “forbidden” transitions, with meaningful consequences for the scope of the present review. The probability of such transitions occurring is significantly lower than that of fundamental transitions (i.e., the most relevant in IR and Raman spectroscopy), which is directly observed as a much lower absorption index of a sample in the NIR region.^[8]^ This results in a deeper penetration of NIR radiation beneath the sample surface (from a few mm to a few cm), giving the possibility of investigating a larger sample volume by means of NIR spectroscopy. Furthermore, the band intensities decrease towards higher NIR wavenumbers, whereas the local‐mode effect makes the spectra relatively simpler.^[9]^ In contrast to IR and Raman spectra, numerous extensively overlapping bands lead to broad line shapes being observed in NIR spectra. Hence, a high spectral resolution of a spectrometer becomes relatively less important in NIR spectroscopy. Furthermore, this peculiarity of NIR absorption bands makes direct interpretation of the spectra more difficult.^[9]^ One should mention the short‐wave NIR (SW‐NIR) region, typically defined as the region at about 14 285–9090 cm^−1^ (700–1100 nm), although the exact boundaries are rather arbitrary in this case.^[10]^ Available technological solutions make it possible to construct very compact and affordable spectrometers operating in this region. SW‐NIR spectroscopy shows great potential and is often used in food analysis.^[11]^ Typically, a very low absorption index (giving the possibility to sense deep beneath the sample surface), suitability of examining moist samples, and good performance in analyzing highly scattering samples should be noted in the context of SW‐NIR spectroscopy in such applications.^[10]^

### 2.2 Basics of instrumentation in NIR spectroscopy

The design of a benchtop NIR spectrometer is in keeping with a general scheme of any instrument used in optical absorption spectroscopy. The main building blocks include a light source, a wavelength selector, and a detector. There are two major classes of such instruments, differing by the principle of how wavelength selection is achieved. Dispersive instruments let only selected wavelengths (narrow waveband) reach the detector at the same time; these wavelengths are selected by, for example, a diffraction grating and optical slit system. The benchtop instrumentation based on this scheme has largely been marginalized by Fourier‐transform (FT) devices with either the most popular Michelson or less common polarization interferometer. FT spectrometers let the entire wavelength region reach the detector in the form of an interferogram, a frequency‐dependent quantity. In principle, this gives a straightforward gain in optical throughput of the spectrometer, resulting in a better signal‐to‐noise (S/N) ratio (SNR). It should be noted, however, that most portable NIR spectrometers use distinctively different technologies and spectroscopic elements to acquire spectra.

### 2.3 Distinctiveness of the design of miniaturized NIR spectrometers

#### 2.3.1 Light sources

In principle, two different types of NIR radiation sources are used in commercially available miniaturized spectrometers. The first one, a tungsten halogen light bulb, is a well‐known standard used in benchtop instruments. It is a thermal radiation source, in which a filament undergoes resistive heating by an electric current passing through it. Through a halogen cycle, tungsten circulates between the filament and halogen gas filling the volume of the bulb. It yields light of high brightness with an intensity and spectral emission profile that depends on the temperature of both the filament and the inner wall of the lamp. Following Planck's law, to stimulate the emission with a peak maximum located in the NIR region, relatively higher temperatures need to be reached than those in the case of IR radiation. A thermal emission source is reliable, inexpensive, and gives a stable output once thermal equilibrium is reached. However, for adoption in miniaturized devices, a number of additional challenges and prerequisites need to be addressed. In addition to the elevated requirement for the source's power efficiency and its physical dimensions, the thermal stability may become an issue. Handheld spectrometers are particularly prone to temperature variations for several reasons. In‐field operation exposes the device to external conditions. Furthermore, compact dimensions reduce the thermal capacity of the device, easing temperature buildup over operation time. The simplest solution recommended by some vendors is to perform frequent reference scans, to keep the background signal most recent. Frequent reference scans are not problematic, in many cases, because these devices often feature rapid scanning. However, in certain applications, such a solution may not be feasible. It has been shown that an insufficient thermal stability negatively influences the analytical performance (MicroNIR 2200).^[12]^ Solutions in the form of thermoelectric cooling have been proposed. On the other hand, some newer devices offer a temperature‐correction function to account for the drift of the emission profile of the source (MicroNIR ES 1700). A tungsten halogen radiation source is employed in, for example, MicroNIR series or microPHAZIR handheld spectrometers.

The second solution, suitable for miniaturized spectrometers, are light‐emitting diodes (LEDs). In principle, a LED is a semiconductor element, in which, upon current flow, recombination of electrons and electron holes occurs and excess energy is emitted as photons.^[13]^ LEDs have several considerable advantages for application in highly miniaturized spectrometers. They feature very compact dimensions, low power consumption, require low voltages for their operation, and are robust and inexpensive. However, there are significant limitations of LEDs as light sources, in general, in spectroscopy. It is primarily a narrow emission bandwidth, for example, a GaAs LED has a maximum emission at 870 nm and a bandwidth of only 50 nm.^[14]^ Furthermore, the availability of LEDs emitting in the NIR region remains very limited. Sources covering the Vis/SW‐NIR region are, however, available and commercially used in miniaturized spectrometers, for which the compact dimensions and affordability are emphasized (e.g., SCiO).

#### 2.3.2 Wavelength selection techniques

The most essential element for a spectrometer is the wavelength selector. Unlike benchtop NIR spectrometers, which are dominated by FT instruments equipped with a Michelson interferometer, portable devices show much diversity here. Instruments based on the principles of a Fabry–Pérot interferometer, Hadamard mask, linear variable filter (LVF), or digital micromirror array are available on the market. Furthermore, some miniaturized designs implement a Michelson interferometer on a microscale.^[4]^ The wavelength selection mechanisms dictate whether a cost‐effective single‐pixel detector (i.e., single spectral resolution element) or a complex array detector needs to be used in the spectrometer. Several of the wavelength selectors could be miniaturized through micro‐electromechanical systems (MEMS; or micro‐opto‐electromechanical systems (MOEMS) if micro‐optics is also included).^[15]^ These optomechanical devices are capable of digital light processing (DLP). MEMS are manufactured in a technology similar to that used for integrated circuitry (microfabrication in silicon).

Despite becoming the standard in benchtop spectrometers, a Michelson interferometer in a miniaturized spectrometer initially faced limitations in implementation. Nonetheless, commercially successful instruments based on this element are available (e.g., NeoSpectra sensors). Additionally, MEMS‐based FTNIR spectrometers face the problem of limited light‐throughput efficiency; however, measures are taken to improve this parameter. A Hadamard‐transform (HT) spectrometer seems more feasible for miniaturization. The design is analogous to that of a grating instrument.^[16]^ In its simplest form of a singly encoded HT spectrometer, the light beam is focused onto a slit and, after passing through a grating and associated optics, it is encoded by a Hadamard mask (a multiaperture mask) and directed to a single‐pixel detector. This optical configuration leads to a Hadamard‐encoded signal reaching the detector, and the spectrum is recovered through HT. The advantages of Hadamard spectrometers were demonstrated in theory relatively early, sharing the optical advantages (multiplex (Felgett), frequency‐precision (Connes), and throughput (Jacquinot)) of FT spectrometry, without the need to use moving parts.[^16^, ^17^] Practical benefits of Hadamard NIR spectrometers were well explained by Fateley and associates.[^18^, ^19^]

A Fabry–Pérot interferometer is equally suitable as a miniaturized wavelength selector. It uses a Fabry–Pérot filter with two parallel mirrors separated by a distance *d*, creating an optical cavity. Either fixed or variable *d* can be used. The optical cavity controls the interference condition through the effect of a standing electric field wave between the two mirrors. Only wavelengths that are in resonance with the optical cavity can pass through the filter. The configuration of the Fabry–Pérot interferometer enables the incident polychromatic light to be divided into several narrower wavelength bands. A fully programmable Fabry–Pérot interferometer can be microfabricated by using MEMS technology. Importantly, such a solution enables the spectrometer to be easily reconfigured to operate over a wide spectral region (e.g., NIRONE sensor series).

A MEMS‐based array of mirrors forms a digital micromirror device (DMD). This element is being used to obtain a microscale dispersive scanning spectrometer with no macroscale moving parts. DMD takes the functional role of a moving dispersion grating. In addition to gains in the cost and size of the wavelength selector element itself, such an optical configuration allows the use of an inexpensive single‐pixel detector.

Wavelength selectors suitable for miniaturized NIR spectrometers can be constructed by using technology other than that of MEMS. A LVF is a notable example. It operates as an optical bandpass filter by using an optical coating of varying thickness across its wedged geometry. This yields a linear change in the transparence of the filter against different wavelengths. A wavelength selector based on a LVF is highly cost‐effective and, in contrast to MEMS, does not involve a very high initial investment cost that is characteristic for semiconductor manufacturing. It is extremely compact and allows spectrometers with very short optical path to be built. Furthermore, no macro‐ or micromoving parts are used, improving the ruggedness of the spectrometer. However, the LVF requires the use of a complicated array detector placed behind the filter; each element of the array creates a separate resolution channel. Notwithstanding, such a multichannel optical configuration provides a good optical throughput and the capacity to achieve a very short time of spectra acquisition.^[3]^

#### 2.3.3 Detectors

Two classes of detector are typically used in miniaturized spectrometers.^[3]^ Photovoltaic Si diodes maintain a suitable sensitivity over the 14 285–9100 cm^−1^ (700–1100 nm) region, and thus, are only suitable for compact, inexpensive spectrometers operating in the Vis and SW‐NIR regions. Furthermore, this solution yields a rather inferior level of S/N. Photodiodes used in portable spectrometers require the use of wavelength cutoff filters to eliminate the risk of the detector responding to sunlight. If mid‐ and long‐wave (LW) sections of the NIR region (i.e., 9500–400 cm^−1^; 1050–2500 nm) are considered, other detectors are indispensable. Because of the need to maintain an adequate S/N in a miniaturized spectrometer, high‐performing InGaAs photodetectors dominate in these devices.^[3]^ The typical range of sensitivity is about 11 100–5882 cm^−1^ (900–1700 nm). Compared with other detectors, InGaAs has a rapid response time, good quantum efficiency, and low dark current at a given sensor area, enabling short scanning times with good S/N. There exists an extended InGaAs variant, suitable for detecting wavelengths longer than 1700 nm; however, it features lower S/N and requires cooling.^[3]^

#### 2.3.4 Optical materials

Optical materials not absorbing in the Vis region are most often transparent throughout a large portion of the NIR region as well. Importantly, this makes glass optics suitable, whereas, for best operation in the LW‐NIR region, high‐quality (i.e., without impurities containing O−H groups) fused silica (fused quartz) optics may be required. This forms a significant advantage for portable NIR spectrometers, making them cheaper and suitable for operation under humid conditions because no alkali halides are used as optical materials. These are critical advantages in on‐site analysis or process monitoring. For rugged operation, a scratch‐resistant optical window at the sample interface is favored for feasible contact‐mode operation. Sapphire is a mechanically durable material used in such a role (e.g., in MicroNIR spectrometer). However, it has the disadvantage of a high refractive index (above 1.7 in the Vis and NIR regions), which increases optical loss due to reflection; this makes the material more suitable for instruments with good optical throughput (e.g., multiplexed or multichannel spectrometers).

Notably, only some miniaturized spectrometers require reflective elements (mirrors) in their design. In this role, gold‐plated surfaces offer superior effectiveness, with a reflectivity of at least 96 % throughout 700 to 2500 nm, that is, the entire NIR region.

#### 2.3.5 Overview of the design of selected handheld NIR spectrometers

**Figure 1:**
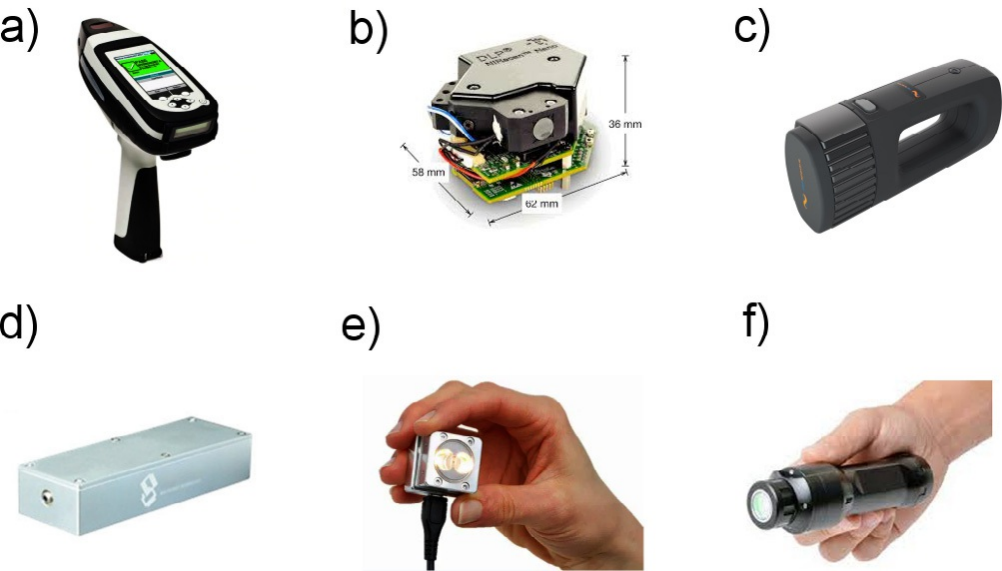
Selected popular miniaturized NIR spectrometers: a) MicroPHAZIR (Thermo Fisher Scientific); b) DLP NIRscan Nano evaluation module (EVM; Texas Instruments); c) NeoSpectra (Si‐Ware Systems); d) nanoFTIR NIR (SouthNest Technology); e) NIRONE Sensor S (Spectral Engines); and f) MicroNIR Pro ES 1700 (VIAVI).

In this and subsequent sections, primary focus is given to the instruments intended for laboratory and on‐site analysis, whereas the designs intended for online applications in industry are mentioned briefly. Although the principal advantages of Hadamard spectrometers were demonstrated early,[^16^, ^17^, ^18^, ^19^] these devices became practically meaningful once a programmable microscale Hadamard mask became implementable through MEMS. This solution was used by the Polychromix company in a NIR spectrometer designed for the exploration of the moon by NASA. Such an application requires a rugged, reliable, and compact instrument. Closely connected to that design was the first handheld NIR spectrometer officially launched in 2006, microPHAZIR (presently, the intellectual property of Thermo Fisher Scientific Inc.; Table 1 and Figure 1 a).^[20]^ This instrument uses a programmable Hadamard mask in the form of a MEMS chip with electronically actuated bars akin to a piano keyboard. It is combined with a low‐power tungsten bulb source and a single‐pixel InGaAs detector. The device offers a rapid scanning capability with a full spectrum recorded under 10 s, good S/N level, and a reasonably good optical resolution of 11 nm. However, the device operates in a rather narrow‐wavelength region of 1596–2396 nm (6267–4173 cm^−1^). MicroPHAZIR is equipped with its own power source (Li‐ion battery, which can be exchanged, and thus a spare battery can be carried for prolonged operation), display screen, and user interface for entirely autonomous operation. Therefore, this device is perfectly suited for on‐site measurements.

**Table 1:** A summary of the technical parameters of the selected popular miniaturized NIR spectrometers.

Spectrometer	Key components			Wavelength region^[a]^		Spectral resolution	S/N^[a]^
(vendor)	source	Wavelength selector	detector	[nm]	[cm^−1^]	(at wavelength)^[b]^ [nm]	
microPHAZIR (Thermo Fisher Scientific)	Tungsten halogen	MEMS Hadamard mask	InGaAs (single element)	1596–2396	6267–4173	11	N/A
MicroNIR Pro ES 1700 (VIAVI)	Tungsten halogen (duplicated)	LVF	InGaAs (array; 128 elements)	908–1676	11 013–5967	12.5 (1000) 25 (2000)	23 000:1
SCiO (Consumer Physics)	LED	bandpass filter	Si photodiode (array, 12 elements)	740–1070	13 514–9346	N/A	N/A
NIRscan (Texas Instruments)	Tungsten halogen	stationary dispersive grating and MEMS DMD	InGaAs (single element)	HP: 1350–2490 MS: 900–1700	HP: 7407–4016 MS: 11 111–5882	HP: 12 MS: 10	HP: 30 000:1 MS: 6000:1
NIRONE sensors (Spectral Engines)	Tungsten halogen (duplicated)	MEMS Fabry–Pérot interferometer	S1.4: InGaAs (single element) S1.7–S2.5: “extended” InGaAs (single element)	1100–1350 (S1.4)^[c]^ 1350–1650 (S1.7)^[c]^ 1550–1950 (S2.0)^[c]^ 1750–2150 (S2.2)^[c]^ 2000–2450 (S2.5)^[b]^	9090–7407 7407–6060 6451–5128 5714–4651 5000–4081	12–16 13–17 15–21 16–22 18–28	15 000:1–1500:1^[b]^ (S1.4–S2.5)
NeoSpectra scanner (Si‐Ware Systems)	Tungsten halogen	MEMS Michelson interferometer	InGaAs (single element)	1350–2500	7407–4000	16 (1550)	N/A
nanoFTIR NIR (SouthNest Technology)	Tungsten halogen	MEMS Michelson interferometer combined with large mirror	InGaAs (single element)	800–2600	12 500–3846	2.5 (1000) 6 (1600) 13 (2400)	9000:1

[a] HP: high performance, MS: mobile sensing. [b] Parameter listed, if available in the data sheet provided by the vendor. [c] Depending on the detector.

The initial success of the microPHAZIR design allowed the anticipation that similar constructions (a Hadamard mask implemented through a MEMS‐based programmable actuator combined with diffraction grating) would rapidly dominate the market of miniaturized NIR instruments.^[3]^ However, further developments progressed differently with the appearance of numerous, distinct designs. A different solution was implemented in the Digital Light Processor (a trademark owned by Texas instruments) NIRscan module from Texas Instruments (Table 1 and Figure 1 b). In this design, a MEMS DMD enables the use of a stationary diffraction grating in a scanning spectrometer. This allows an expensive microarray detector to be replaced by a large single‐pixel detector. Thus, a very simple, cost‐effective construction of a miniature dispersive instrument was achieved. The low power consumption of such a design should also be noted. These spectrometers offer spectral resolution and sensitivity that is adequate for certain applications. The developed technology is available commercially as two EVMs: HP EVM featuring a DLP NIRscan sensor and a MS EVM with a DLP NIRscan Nano (Table 1).^[21]^ Although the larger DLP NIRscan sensor is advertised as being suitable for integration into spectrometers intended for both in‐field and industrial online use, the compact NIRscan Nano module is suited for portable spectrometers. The latter solution is implemented in a NIR‐S‐G1 instrument offered by InnoSpectra^[22]^ and available as a customized product from SphereOptics,^[23]^ Sagitto,^[24]^ Allied Scientific,^[25]^ and Tellspec.^[26]^ The NIR‐S‐G1 spectrometer has extremely compact dimensions (82×63×43 mm^3^; <145 g weight) and uses a tungsten halogen source and InGaAs detector. The working spectral region of the device (11 111–5082 cm^−1^; 900–1700 nm) covers a wide part of the NIR wavelengths. The spectrometer is controlled through a mobile application and communicates with a smartphone over a low‐power wireless (Bluetooth) interface.

Miniaturized FTNIR spectrometers with a Michelson interferometer were commercialized by Si‐Ware Systems in their NeoSpectra device (Table 1 and Figure 1 c).^[27]^ As explained in Section 2.3.2, this particular principle of operation is difficult for miniaturization without performance penalties. However, continuous development has focused on improving the operation of the interferometer and the optical throughput of such devices should be noted. For example, the nanoFTIR NIR spectrometer was announced last year by Hefei SouthNest Technology (Table 1 and Figure 1 d).^[28]^ The device uses a MEMS Michelson interferometer with a large mirror (relative to the area of the MEMS chip) to enhance its optical throughput. Notably, the spectrometer operates over the entire NIR region (12 50–846 cm^−1^; 800–2600 nm) with a relatively high spectral resolution (6 nm at 1600 nm). The instrument has compact dimensions (14.3×4.9×2.8 cm^3^) and low weight (220 g). Rugged build and fiber‐probe connectivity of the instrument make it particularly suitable for online analysis. A new MEMS‐based FTNIR spectrometer was recently introduced by Hamamatsu as well.^[29]^ To overcome optical throughput limitations, the device features a 3 mm in diameter moving mirror, resulting in a high S/N over the wide spectral region of 1100–2500 nm (9090–4000 cm^−1^). The device is offered as a compact spectrometer module (ca. 300 g of weight), with USB connectivity and a fiber‐probe interface.

A tunable Fabry–Pérot interferometer miniaturized as a MEMS is used in the NIRONE Sensor S instrument (Table 1 and Figure 1 e) available since 2016.^[30]^ Despite very compact dimensions (25×25×17.5 mm^3^; 15 g weight), the sensor is equipped with two tungsten halogen lamps and a single‐element detector. Several variants of the sensor are available that differ by the operational wavenumber region, resolution, and S/N parameter (Table 1). Implementation of the Fabry–Pérot interferometer enabled an optical configuration of the sensor suitable for a relatively large area, with either an InGaAs or an extended InGaAs detector; the former type yields S/N up to 15 000:1, granting a reasonable level of sensitivity and specificity of the instrument. Sensor X is a scaled‐down variant, optimized for cost‐effectiveness and ease of manufacturability. Importantly, current advances in the technology of Fabry–Pérot interferometers are promising with regard to ultraminiaturization. Hamamatsu recently introduced an ultracompact NIR sensor that integrated a Fabry–Pérot interferometer through MEMS technology and operated in the 1550–1850 nm (6452–5405 cm^−1^) region.^[31]^ The sensor is hermetically sealed for high reliability under high humidity, while remaining extremely compact (<1 g of weight).

A radically different design approach was employed in the MicroNIR series of instruments from VIAVI (Table 1 and Figure 1 f). This compact spectrometer uses a complex and more expensive array detector (InGaAs) combined with a LVF element. This yields an extremely small and robust device, with no moving parts and reliable operation under difficult conditions; similar technology is used in sensors aimed at process control. Newer variants of a MicroNIR instrument improve the stability of the operation time by implementing a temperature‐correction function. The optical configuration of this multichannel device enables rapid measurement and good quality of the spectra. The MicroNIR 1700 device is the variant powered and controlled by a USB interface, and therefore, during on‐site use, it requires continuous connection to a master device (e.g., notebook PC). A more convenient version of the sensor, MicroNIR OnSite‐W, is equipped with its own battery power source and water‐ and dust‐resistant case, making it even more suited for on‐site use.^[32]^ VIAVI offers a similar design in their line of spectrometers intended for online and inline monitoring of processes in the chemical, pharmaceutical, and food industries. MicroNIR PAT analyzers are offered in three variants (MicroNIR PAT‐U, PAT‐W, and PAT‐Ux), in which different features required in the applications are accommodated, for example, an increased ruggedness, resistance to environment (e.g., water, dust, chemical agents), fiber‐probe connectivity or sanitary (hygienic) flange mounts, and control software designed for such uses.^[33]^ A number of dedicated portable NIR sensors intended for online use are available, for example, Axsun IntegraSpec XL NIR from Excelitas Technologies,^[34]^ or a mobile unit (mounted on a movable cart and equipped with its own power source) MB3600‐PH FTNIR spectrometer from ABB.^[35]^ Interestingly, the Axsun instrument incorporates MEMS tunable laser technology, yielding fast measurement speed, high spectral resolution, and good S/N, which are helpful in process monitoring applications.^[34]^

Some of the extremely compact and cost‐effective NIR spectrometers have been engineered by accepting a somewhat limited general applicability and performance in general use, yet adequate suitability for certain applications. A good example of such an instrument is the SCiO NIR microspectrometer from Consumer Physics, which has been available since about 2015.^[36]^ Advertised as the first pocket‐sized spectrometer, the device has dimensions of 67.7×40.2×18.8 mm^3^ and a weight of 35 g, and is aimed at the consumer market. Unparalleled affordability was achieved through the use of a LED light source and a simple Si photodiode array detector (4×3 configuration), with optical filters over the individual pixels creating a 12‐channel device. However, noticeable penalties to the performance of the spectrometer have been unavoidable in such a design. A subpar S/N, very narrow Vis/SW‐NIR operating wavelength region (13 514–9346 cm^−1^; 740–1070 nm), and poor spectral resolution of about 28 nm because of a low number of resolution elements should be noted. The product is addressed for consumer use, and therefore, a compromise in the performance in exchange for affordability seems justified.

It should be noted that, in research laboratories and in many applications, the calibration is generated by the operator, often by using software different from that provided by the vendor of the device. Although the vast majority of miniaturized instruments may operate as such general‐purpose NIR spectrometers, a number of them are also sold preconfigured with calibrations prepared for intended analyses. Their operations do not require in‐depth knowledge about spectroscopy or data‐analytical methods, and satisfactory analyses may be performed by untrained personnel. Such dedicated analyzers are becoming popular in regular use, particularly in agriculture. For instance, NIR4 Farm, available from AB Vista, is a portable spectrometer intended for forage analysis.^[37]^ Unlike the common time‐consuming way of shipping samples to a commercial laboratory, a direct on‐farm analysis enables quick optimization of the ration and maximizes milk yield. Other conceptually similar products should be mentioned as well, for example, the AURA handheld NIR^[38]^ or X‐NIR analyzer,^[39]^ which are both preconfigured for the assessment of grain, or slightly larger, portable in a suitcase format, StellarCASE Portable NIR analyzer, intended for material analysis.^[40]^ Comparable concepts exist for sensors intended for online use, for instance, a nondrift NIR analyzer from MoistTech Corp.^[41]^

Notably, some of the instruments on the market are available either as a general‐purpose spectrometer or in a preconfigured setup. For instance, microPHAZIR has been sold as an analyzer for animal feed and ingredients, with several calibrations prepared for such a scope.^[42]^ Offered as the microPHAZIR AG handheld analyzer, the device is intended for ease of use, with the ability to estimate the key components in feed (e.g., moisture, protein, fiber, starch). Other turnkey preconfigurations of the same hardware should be mentioned as well, for example, a plastics recycling analyzer (microPHAZIR PC),^[43]^ a pharmaceutical analyzer (microPHAZIR RX),^[44]^ and an asbestos analyzer (microPHAZIR AS).^[45]^

The current development trend aims for further ultraminiaturization to the extent of enabling implementing a NIR spectrometer directly in smartphone devices.^[46]^ Such designs have already been announced, although not yet available as the final product, indicating that certain challenges have not yet been fully overcome. It should be briefly mentioned that there are spectrometers mountable in airborne drones (unmanned aerial vehicles (UAVs)), which can be considered a highly specialized type of portable NIR spectrometer. The development of such technology faces similar challenges and shares similar goals with the topic reviewed herein. Notably, UAV‐borne spectral sensors are currently mostly used for multispectral imaging.^[47]^ Quantitative analysis of several properties of crops was shown to be feasible based upon acquisition of narrow spectral bands from UAV‐borne spectrometers, additionally compared in performance with ground‐based instrument.^[48]^ In some cases, higher resolution spectral information covering the NIR region is processed.^[49]^ The advances in sensor technology and data‐analytical tools, as well as studies aimed at resolving fundamental issues, such as reliable direct measurement of reflectance,^[50]^ form important steps in fully suited airborne NIR spectroscopy.

#### 2.3.6 Principles, treatment, and consequences of the instrumental differences in the context of portable spectrometers

Instrumental difference is a well‐known phenomenon, in which even very subtle dissimilarities are observed between the spectral line shapes measured on various spectrometers for the same sample and under identical external conditions. It is apparent for benchtop FTNIR instruments and leads to nonstraightforward transferability of calibration models. In other words, calibrations developed on a given instrument, most of the time, cannot be used for the analysis of unknown samples on any other spectrometers. Because calibration is a time‐ and resource‐consuming process, calibration transfer procedures were intensively researched.^[51]^ Compared with conventional instruments, diversity in the technical principles underlying microspectrometers goes much further, as presented in Section 2.3.5. Nonidentical wavelength resolutions, spectral sensitivity, and S/N levels strongly contribute to instrumental differences. As may be concluded from the information provided in Table 1, miniaturized spectrometers strongly vary in these regards. Therefore, their distinct operational characteristics may lead to highly elevated instrumental dissimilarities. Furthermore, decisive differences between the wavelength regions that these devices can measure may directly determine the applicability to certain types of samples. For instance, in food analysis, the SW‐NIR region is considered to be suitable for macronutrient determination in moist samples or water‐content analysis. However, one may not be able to measure weak absorption of less abundant chemicals in the SW‐NIR region with adequate accuracy. This enables the suitability of SW‐NIR spectrometers for certain purposes to be roughly estimated. On the other hand, differences between instruments, in their capacity to analyze the LW‐NIR region, are more difficult to predict and require more systematic approaches.^[52]^

The current state of technology only requires a miniaturized NIR spectrometer to be a balance between the level of miniaturization, performance, and economic cost. Furthermore, this type of instrumentation continues its rapid development, with new spectrometers pushing the envelope in operational characteristics, for example, mini‐FTNIR devices capable of operating over the entire NIR wavelength region (Table 1). Therefore, the applicability of these devices in various scenarios, as well as their analytical performance, is an intensively explored area.^[4]^

## 3 Miniaturized NIR Spectroscopy in Practical Applications

### 3.1 Major fields of application

#### 3.1.1 Pharmaceuticals

Pharmaceutical analysis is a particularly demanding scenario for analytical techniques, in which accuracy and reliability are foremost important. Nonetheless, flexibility for on‐site measurements is a considerable gain for the pharmaceutical industry as well, and therefore, miniaturized NIR sensors have attracted keen attention. The current state of industrial applications of NIR spectroscopy have been thoroughly described by Chavan et al.^[53]^ It was pointed out relatively early that the applicability and performance of handheld NIR instruments for qualitative and quantitative analyses of pharmaceutical formulations require systematic feasibility studies. Subsequent examinations revealed that certain miniaturized devices could offer competitive levels of accuracy and reliability, for example, as demonstrated by Alcalà et al.^[54]^ for MicroNIR spectrometers. Two versions of this device were used: the standard one operating in the 10 526–6060 cm^−1^ (950–1650 nm) range and variant with an extended InGaAs detector that covers the 8695–4651 cm^−1^ (1150–2150 nm) range. Qualitative applications, that is, classification, have wide practical importance because of drug counterfeiting, illegal drug imports, and online drug trading.[^54^, ^55^, ^56^, ^57^] Portable NIR spectrometers offer great potential for rapid analyses performed directly by border control or postal staff, largely improving the throughput of the control procedures. Qualitative analysis is, most of the time, less sensitive to the instrumental factors and could be accurately performed with a MicroNIR device.^[54]^ First, the identification of raw materials commonly used in pharmaceutical formulations could be performed by means of routinely used methods (e.g., principal components analysis (PCA)), as demonstrated on the basis of 22 compounds. False‐positive and near‐false‐positive identifications occurred only among chemically similar materials. Furthermore, successful discrimination between authentic and counterfeit drugs with 95 % confidence was accomplished for several pharmaceutical products (Alli®, Viagra®, Cialis®) and their illegal counterfeits, which were analyzed as tablets and capsule blends. Furthermore, quantitative determination of active pharmaceutical ingredients (APIs), in this case, acetylsalicylic acid (ASA), ascorbic acid (ASC), and caffeine (CAF), in pharmaceutical formulations in powdered form was performed. Accurate determination of the concentrations of active principal ingredients in unknown test samples could be performed through partial least squares regression (PLS‐R) models.

**Figure 2:**
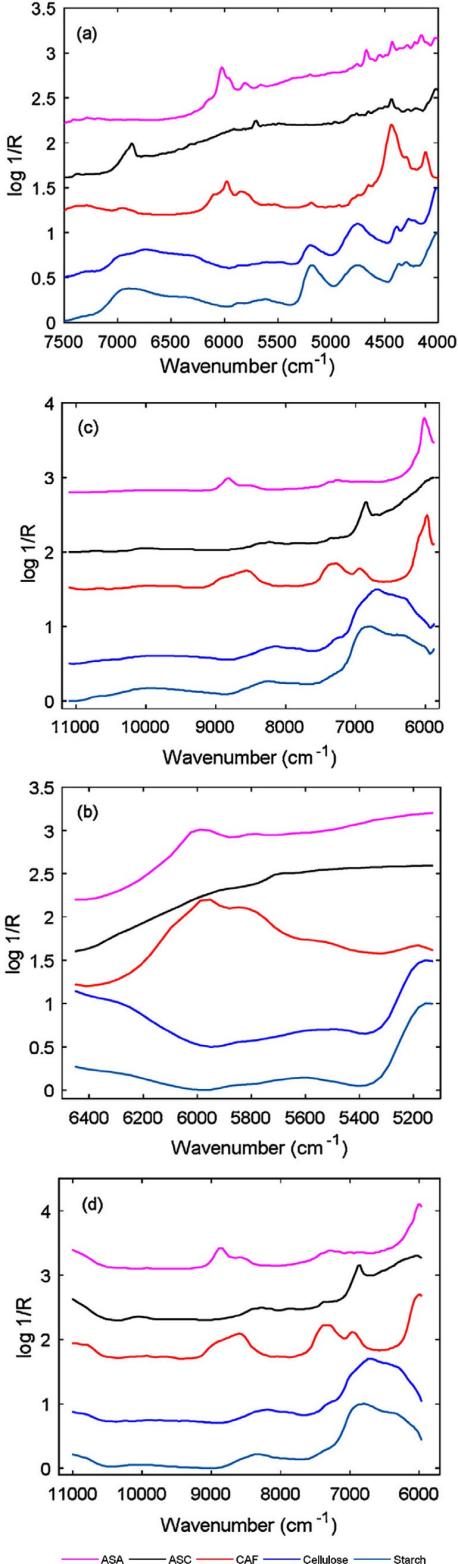
NIR spectra of the pure ingredients of the pharmaceutical formulation investigated by Yan and Siesler^[58]^ recorded with the miniaturized spectrometers: a) NeoSpectra, b) NIRONE, c) DLP NIRScan, and d) MicroNIR. Reproduced with permission from ref. [58]. Copyright Elsevier, 2018.

Direct comparisons of the applicability and performance of different miniaturized NIR spectrometers in analyzing pharmaceutical formulations were performed. For example, recently, Yan and Siesler examined four instruments that used distinct design principles: NeoSpectra, NIRONE (Spectral Engines NR‐2.0 W), DLP NIRscan, and MicroNIR.^[58]^ Consequently, the captured spectral information differs notably between these instruments, as presented in Figure 2. The subjects for quantitative analysis were two excipients (cellulose and starch) and three APIs (ASA, ASC, CAF). The study indicated that the prediction performance of these four instrument varied for the studied analytes, with the exception of CAF, in which case all devices performed comparably well. The conclusions drawn indicated that the LVF/InGaAs array spectrometer (MicroNIR) yielded the most balanced performance, with the DMD‐based device (NIRscan) following closely behind. The remaining two spectrometers performed notably worse, although still offered prediction capabilities acceptable for quantitative analysis in the investigated scenario. This provides evidence for the performance penalties of miniaturized interferometer‐based spectrometers (in that study, NeoSpectra with a Michelson interferometer and NIRONE with a Fabry–Pérot interferometer) clearly translating into their practical applicability.

In a recent study by Guillemain et al.,^[59]^ in addition to the routinely used unsupervised method PCA, a number of supervised classification techniques (linear discriminant analysis (LDA), quadratic discriminant analysis (QDA), support vector machine, SVM; *k*‐nearest neighbors (KNN)) were evaluated in tablet authentication from the spectral measurements performed with two different NIR miniaturized spectrometers (MicroNIR and SCiO). The compared instruments differ notably in their design and operational characteristics, foremost, in the measured spectral region (Table 1). Interestingly, despite being extremely affordable, the SCiO instrument performed better in this application. For each of these two instruments, different classification methods were concluded to be the best performing ones: SVM for SCiO and LDA for MicroNIR. The classification performance for 29 pharmaceutical products was reported to be 100/96 (% of correct identification in calibration/in validation) in the former and 99.9/91.1 in the latter. This suggests that spectral information relevant for the authentication of the pharmaceutical formulations investigated in that study was relatively more accessible in the SW‐NIR region (over which the SCiO instrument operates; Table 1).

The development of applications of miniaturized NIR spectrometers in the quantitative determination of APIs continues. A focus on improving their performance in more demanding scenarios can be observed. For example, a recent study aimed at the simultaneous quantification in a tablet of two APIs (paracetamol and tramadol), which are spectrally similar and present in the sample in greatly different mass proportions.^[60]^ Despite these difficulties, a handheld NIR spectrometer (MicroNIR) performed comparably well to that of a benchtop spectrometer. However, it should be noted that preselection of the spectral region for subsequent analysis was needed in this case.^[60]^ The need for applying different pretreatments to the spectra acquired by various spectrometers, for example, as the result of poorer spectral resolution of S/N, should be mentioned, but such a step does not significantly complicate the flow of the analysis.^[54]^ Often, additional reasons for extended supervision result from the pronounced differences between the spectral regions measured by various miniaturized instruments. For instance, a comparative study reported that different classification methods maximized the accuracy of counterfeit detection in the case of pharmaceutical tablets performed by two portable instruments.^[59]^ In the case of microPHAZIR, which operates in the conventional NIR region, the application of LDA was deemed to be more successful. In contrast, SVM classification yielded the best results for data collected with the SW‐NIR SCiO spectrometer.^[59]^

These studies indicated that, in the case of miniaturized NIR spectrometers, more attention should be given to the careful selection of spectral pretreatments and chemometric methods for subsequent analysis.[^54^, ^59^, ^60^] The importance of proper preprocessing confirms that certain limitations of compact instruments, for example, narrow observed spectral regions, SNR, or spectral resolution, need to be taken into account more carefully than in the case of benchtop spectrometers. In some cases, a higher requirement of sample preparation for successful analysis by miniaturized spectrometers was reported as well. For instance, milling of plant material may significantly improve the performance of the analysis.^[52]^

Notably, handheld NIR spectrometers face competition from Raman instruments in applications, in which cost‐effectiveness does not play a crucial role. The strengths and limitations of both techniques in the analysis of pharmaceutical products were directly compared, for example, in a recent study by Ciza et al.^[61]^ The general conclusion was that miniaturized NIR spectroscopy was more reliable in specific product identification; however, the choice of a better technique might depend on the particular application. It was suggested that the future appearance of cost‐effective instruments combining both NIR and Raman spectrometers would result in substantial progress.^[61]^ A wider perspective of how portable NIR spectrometers compete with Raman instruments in the analysis of pharmaceuticals has been presented in a recent review article.^[62]^

#### 3.1.2 Natural medicines

Despite apparent similarities to the field discussed in Section 3.1.1, the purpose and characteristics of the analyses performed on natural medicines (i.e., plant extracts or fresh plant materials) differ in several aspects. Natural products are incomparably more complex in their chemical composition (i.e., leading to considerable matrix effects), often with the active compound being in a relatively low concentration, the chemical and physical properties of the sample vary from batch to batch and may be affected by the sample preparation procedures. Although these factors increase the difficulty of the analysis, they also make miniaturized NIR spectroscopy particularly important for its capacity to perform rapid and high‐throughput quality control of such products.

**Figure 3:**
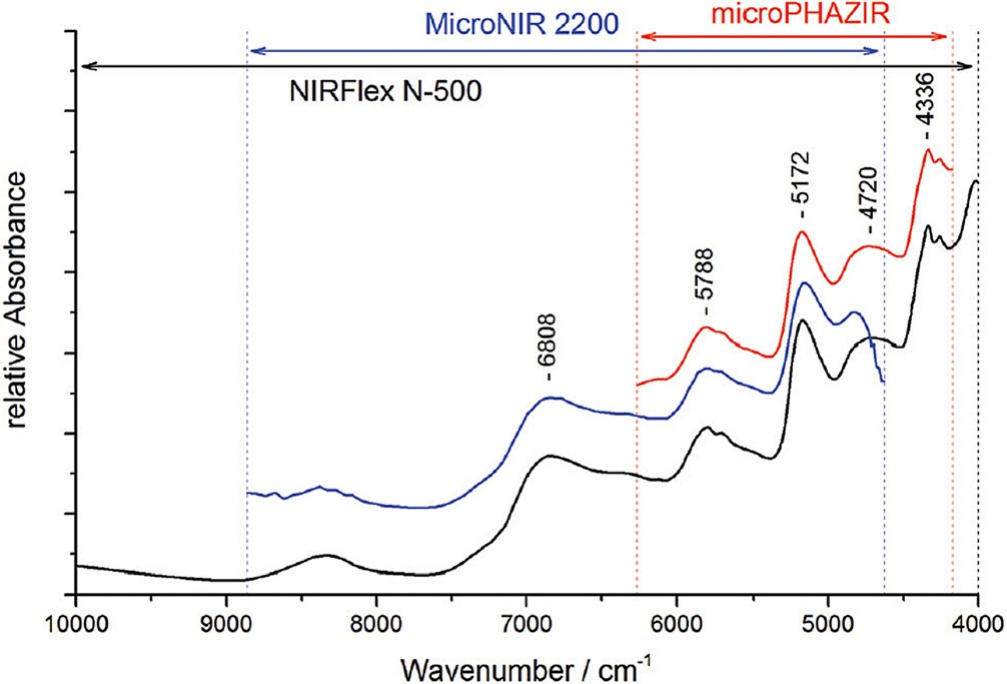
NIR spectra of 60 *R. folium* samples. Reproduced with permission from ref. [63]. Copyright Royal Chemical Society, 2017.

Miniaturized NIR spectrometers can be successfully used for quantitative determination of the active compounds in natural medicines, for example, as shown by Kirchler et al.^[63]^ The performance of two handheld NIR spectrometers (MicroNIR and microPHAZIR) in analyzing 60 samples of extracts from rosemary leaves (*Rosmarini folium*) was compared with the results obtained from a benchtop instrument (Büchi NIRFlex N‐500). The analysis was directed at the quantitative determination of polyphenolic content, mostly contributed to by rosmarinic acid (RA), in the medicine. Instrumental difference was examined, in detail, in the study, including the clear disparity in the covered spectral regions (Figure 3). The study included a detailed analysis of the vibrational transitions of RA, yielding a detailed overview of which vibrations these portable instruments can observe and how relevant the corresponding wavenumbers are in PLS‐R models of NIR spectral features associated with RA content in the studied samples. This enabled a deeper insight into the instrumental differences to be obtained. Further insights into the assessment of the relative sensitivity of each spectrometer towards the absorption regions associated with specific molecular vibrations were provided by quantum mechanical simulations of the NIR spectrum of RA and two‐dimensional correlation analysis (2D‐COS). The quantitative analytical performance of the three evaluated spectrometers indicated that, although the best results were provided by the benchtop NIRFlex N‐500 instrument, among the miniaturized devices, the efficacy of MicroNIR was also satisfactory and superior to microPHAZIR in that particular application. The study revealed that the wider observed spectral region, presenting more relevant vibrations of RA to the regression procedure, led to better results of the former device. At the same time, the benchtop spectrometer outperformed both portable devices, although all three yielded performance levels that were satisfactory for quantitative analysis. Notwithstanding, the study confirmed that the physical and chemical properties of plant extracts might pose increased difficulties for the successful application of miniaturized NIR spectrometers.

It is of particular note that portable NIR spectrometers can be used with great success for highly specific purposes in phytopharmaceutical applications. Through direct on‐site measurements on pharmaceutically relevant fresh plant material, a rapid determination of the ideal harvest time of a medicinal plant may be performed. Optimization of the conditions of cultivation towards the best quality (e.g., highest concentration of the active therapeutic ingredient in plant tissues) becomes possible, with significant economic gain for the industry. An example of a feasibility study that demonstrated the emerging potential for such applications may be served by Pezzei et al.^[64]^ Their case study involved the evaluation of the applicability of a handheld NIR spectrometer (microPHAZIR) in predicting an optimal harvest time of a medicinal plant, *Verbena officinalis*, with reference analysis carried out through HPLC with a diode‐array detector based on mass spectrometry (HPLC‐DAD‐MS) and reference NIR spectral analysis performed on a benchtop FTNIR instrument. The optimal harvest time of the plant was related to the concentration of the main therapeutic ingredients, verbenalin and verbascoside. Despite predictions, the power demonstrated by the handheld spectrometer in absolute terms was inferior compared with that of the benchtop instrument. The study confirmed that such a performance level was adequate for this particular application. Hence, miniaturized NIR spectrometers deployed on‐site for direct measurements of fresh plant material offer great potential for guaranteeing a consistently high phytopharmaceutical quality of herbal raw materials.

#### 3.1.3 Agri‐food sector

Agriculture‐ and food‐related analysis is a particularly rich field of application of miniaturized NIR spectrometers. Portable NIR spectroscopy is a perfect tool for monitoring crop quality to determine optimal cultivation conditions. The importance of controlling food quality, as a result of the generally high vulnerability of foods to content variation, maintaining freshness to prevent the risk of quality loss, and possibility of illegal adulteration, and so forth, have led to the wide adoption of NIR spectroscopy as a nondestructive, rapid, and high‐throughput analytical method. Furthermore, the complex nature of food production and delivery chain, as well as the necessity to reduce the analysis time to a minimum, have contributed to emergent portable spectrometers being a revolutionary step forward in this field. The particular exposition of food products to safety risks of various kinds and the key role of conventional NIR spectroscopy as a tool for controlling food safety and quality have been discussed in detail by Qu et al.^[65]^

Portable instruments were successfully validated in food analyses relatively early, as, for example, summarized in 2013 in review articles by Teixeira dos Santos et al.^[66]^ and Alander et al.^[67]^ A perspective view on the particular potential offered by portable NIR instruments and the feasibility of their use throughout the food supply chains has been presented by Ellis and co‐workers.^[68]^ The majority of studies focus on establishing successful methods of determining the quality parameters of final food products. The great variety of chemical (e.g., complex matrix, high moisture content) and physical (e.g., surface texture) properties of such products and often the need to perform analyses in an entirely nonintrusive manner (i.e., through the original packaging) appears. These factors increase the difficulty of such analyses, and the applicability of certain instruments greatly varies, depending on a particular case. Nevertheless, portable NIR spectroscopy has been adopted with great success in the food sector and application development continues to be an active research direction, with numerous reports appearing in the recent literature. Table 2 summarizes current progress being made in this direction. An overview of current progress in food analytical applications of miniaturized NIR spectrometers reveals that, while compact devices can be successfully adopted for numerous types of analyses, the applicability potential and relative performance between devices largely varies.[^126^, ^127^, ^128^, ^129^, ^130^, ^131^, ^132^, ^133^, ^134^, ^135^, ^136^, ^137^] The instrumental differences seem to be particularly evident in such applications, for example, highly affordable SW‐NIR spectrometers may yield good performance in analyzing some macronutrients, yet their applicability is largely limited in other scenarios. This makes it difficult to anticipate the performance of a given spectrometer without prior systematic feasibility studies. Recently, a successful attempt to obtain more general insights into instrumental differences, expressed through the ability to analyze chemical contents with significant differences in their NIR absorption spectra, was conducted by Mayr et al.^[52]^ Differences in the prediction of CAF and l‐theanine contents (considered the quality parameters) in black tea by microPHAZIR and MicroNIR instruments could be associated with their sensitivity towards characteristic absorption bands of these two constituents. Furthermore, spectral simulations yielded a detailed correlation between the absorption bands and structural features of these molecules. Hence, the wavenumber regions meaningful for NIR analysis performed with both devices, which, in this case, largely differed in their operational spectral regions, could be established. These accomplishments enabled the performance of each of these two spectrometers to be predicted in analyzing compounds similar to either CAF or l‐theanine.^[52]^

**Table 2:** An overview of recent research activities in the application of miniaturized NIR spectrometers in food analysis.

Ref.	Scope	Subject/sample type	Miniaturized NIR instrument	Pretreatment and chemometrics	General conclusions (applicability/performance)
[126]	evaluation of benchtop vs. portable NIR instruments in qualitative and quantitative analysis of main sugars in syrup formulations	116 samples (53 standard and 63 reformulated syrups)	microPHAZIR	PCA,^[a]^ PCR, SVMR, PLS‐R	good performance of microPHAZIR over a wide range of sugar concentrations; suitable for practical use in quality control in industry
[127]	feasibility of miniaturized NIR spectrometer to quantify main carbohydrate contents in syrup; evaluation of the potential for consumer use	116 syrups consisting of different flavor types	microPHAZIR	PLS‐R	reliable and accessible use of miniaturized NIR spectrometers by consumers requires further development of robust spectral processing methods that require no/minimal supervision
[128]	performance evaluation of a miniaturized NIR spectrometer in classification of food powders	8 visually indistinguishable food powders: sugar, salt, cream, flour, corn, rice, bean, and potato powder	Link Square (Stratio, Inc.)	SVM, KNN, RF	successful classification of food powders by miniaturized NIR spectroscopy
[129]	performance evaluation of portable NIR spectrometer in authentication of organic milk	87 samples (full‐fat, pasteurized retail milk), including 7 organic retail milks and 50 nonorganic retail milks	MicroNIR	PCA, PLS‐DA	accurate discrimination between organic and conventional milk; less successful class assignment for pasture milk samples; in both cases, MicroNIR was noninferior to the benchtop NIR spectrometer
[130]	performance comparison of benchtop vs. miniaturized NIR spectrometer in detecting meat fraud	63 samples of different meat types (beef: 9, chicken: 10, mutton: 10, turkey: 10, pork: 10, horse meat: 14)	microPHAZIR	PCA, PLS‐R	high‐level meat adulterations (>10 %): fully feasible with benchtop spectroscopy, improvements required for miniaturized instrument (e.g., larger sample set); low‐level meat adulterations (<10 %): improvements are needed for both types of instrumentation
[131]	feasibility study of miniaturized NIR spectrometer in determining nutritional parameters of pasta/sauce blends	commercial products: 5 pasta products, 5 sauce products; for each, 5 different pasta/sauce‐type blend combinations (0–100 % (w/w) sauce addition)	MicroNIR	PLS‐R	satisfactory prediction accuracy for quantifying energy, carbohydrate, fat, fiber, protein, and sugar in pasta/sauce meal through miniaturized NIR spectroscopy in a realistic analytical scenario
[132]	feasibility study for miniaturized NIR spectroscopy in analyzing matcha tea quality index	105 samples of matcha tea samples of different grades	NIRscan Nano	PLS‐R, Si‐PLS, GA‐PLS, CARS‐PLS, RF‐PLS	a model strategy based on portable NIR spectroscopy was successfully developed, with promising potential for predicting and classifying the content of polyphenols and amino acids in matcha tea
[133]	performance comparison of benchtop vs. miniaturized NIR spectrometers in determining quality parameters of cheese	46 cheese samples (20 of hard cheese, 26 samples of semihard cheese, with respect to water content)	SCiO	PLS‐R	good accuracy of a miniaturized, extremely cost‐effective NIR spectrometer in analyzing quality parameters of cheese; acceptable performance, even if using consumer‐targeted software
[134]	performance assessment of a miniaturized NIR spectrometer (NIRscan) for IMF prediction in comparison with two portable and one Vis/SW‐NIR spectrophotometers	IMF determination; frozen (609 samples) or fresh (60 samples) lamb	Labspec5000 Trek (ASD Inc., Boulder, CO, USA), NIRscan Nano	MSC and PLS‐R; VIP analysis for determining the important spectral features (relevant peak related to intramuscular fat) captured by the NIRscan Nano device	prediction performance not affected by sample temperature equilibration time; frozen samples: good performance of LabSpec5000, LabSpec4, and Trek instruments; bias (measurement time‐wise) observed for NIRscan Nano (instrumental variations); fresh meat: NIRscan Nano performed well and is a good alternative to handheld spectrophotometers for rapid and real‐time classification of fresh lamb
[135]	feasibility of miniaturized NIR spectrometer for rapid assessment of pork freshness	80 samples with 4 groups of 20 (storage at 4 °C for 2, 4, and 8 days)	MicroNIR	PLSR, MLR, SPA, RC	good performance of miniaturized NIR spectrometer; suitability for nondestructive monitoring of thiobarbituric acid reactive substances in minced pork
[136]	feasibility study of miniaturized NIR spectrometer in rapid authentication of adulterated paprika powder	3 types of paprika (sweet, smoked, and spicy): 315 samples; spiked with potato starch and acacia gum (0–36 % w/w), and annatto (0–18 % w/w)	NIRscan Nano	PLS‐DA, PLS‐R	good accuracy of NIRscan Nano in detecting adulterated samples and in differentiating types of adulterations (lower only for annatto, yet still adequate for screening purposes)
[137]	authentication of chicken meat by miniaturized NIR spectrometer	153 fresh chicken fillet samples	MicroNIR	PLS‐DA, CP‐ANN, SVM, RSDE	miniaturized NIR spectroscopy provides cost‐efficient, rapid (<20 s for complete analysis), and reliable tool for monitoring meat authenticity (and quality) directly in the field

[a] CARS‐PLS: competitive adaptive reweighted sampling partial least squares; CP‐ANN: counterpropagation artificial neural network; GA‐PLS: genetic algorithm partial least squares; IMF: intermuscular fat; MLR: multilinear regression; MSC: multiplicative scattering correction; PCR: principal component regression; PLS‐DA: partial least squares discriminant analysis; RC: regression coefficients; RF: random forest; RF‐PLS: random frog partial least square; RSDE: random subspace discriminant ensemble; Si‐PLS: synergy interval partial least squares; SPA: successive projection algorithm; SVMR: support vector machine regression; VIP: variable importance in projection.

Importantly, food analysis towards proving its quality, origin, authenticity, or nutritional values attracts great interest from consumers. In this direction, the barrier between professional and causal use of NIR spectroscopy has already been broken. Inexpensive SW‐NIR spectrometers intended to be operated through a “black‐box” application installed on a smartphone are already available and advertised on the market.^[4]^

Agricultural investigations focus on analyzing raw products, with an extensive literature record corresponding to the qualitative and quantitative analysis of crops, fruits, and vegetables. An extensive amount of literature reports on applications of portable NIR spectroscopy aimed in this direction are available because miniaturized NIR spectrometers were widely adopted in agricultural applications relatively early.^[66]^ Often, the methods and general features of such analyses resemble those described in the paragraph above. Considerable attention has been given to fruit analyses by portable NIR spectroscopy.[^69^, ^70^, ^71^, ^72^, ^73^] Among others, the capacity of compact spectrometers to successfully determining quality parameters,^[69]^ monitoring moisture content and controlling drying processes,[^70^, ^73^] determining and authenticating fruit varieties,^[71]^ as well as quantitatively determining various properties of fruits[^70^, ^71^, ^72^] were demonstrated in the recent literature. Similarly high attention was given to the advantages of portable NIR spectroscopy in the analysis of grains. Recent examples of such applications include, for example, the quantitative determination of the total antioxidant capacity in various gluten‐free grains.^[74]^ The study compared the performance of three different portable NIR instruments (microPHAZIR, MicroNIR and SCiO) versus a benchtop spectrometer. The best results were achieved upon measuring nonmilled samples with the MicroNIR instrument, although all evaluated spectrometers performed satisfactorily in this application. The SCiO device performed consistently slightly poorer; however, its high affordability should be stressed. On the other hand, protein analysis in grains proved to be more challenging for miniaturized spectrometers, particularly for nonmilled samples.^[75]^ The suggested reasons for poorer performance may include higher susceptibility to detrimental effects of scattering at the sample surface. This coincides with the observations made in an earlier study, in which sample milling led to a more pronounced performance improvement of portable spectrometers.^[74]^ Those conclusions seem to indicate that, in certain applications, portable spectrometers may require more sophisticated sample preparations.

The potential for nonintrusive examinations of entire seeds by NIR spectroscopy seems to be well‐recognized in agriculture‐related studies. Miniaturized spectrometers were recently used for analyzing whole canola seeds,^[76]^ coffee beans,^[77]^ barley, chickpeas, and sorghum.^[78]^ A number of other ongoing studies have demonstrated portable NIR spectroscopy to be a suitable tool for the quantitative and qualitative examination of various other agricultural products, for example, cherry tomatoes,^[79]^ mulberry leaves,^[80]^ grapes, and peaches.^[81]^

It is noteworthy that NIR spectroscopy is a suitable tool for the analysis of soil and water, and portable instruments markedly improve the ability to perform such monitoring on a large scale. Such a scope also fits the topic of environmental studies, which are discussed jointly in Section 3.1.4. Currently, revolutionary concepts and novel technology lead to the era of precision agriculture.^[82]^ A major role in precision agriculture is played by spectral methods of analysis and, in particular, remote sensing. This technique is based on miniaturized spectrometers mounted on UAVs (that is, airborne drones), enabling remote sensing of large areas of agricultural crops.[^47^, ^48^] The solutions employed are based on specialized technology. However, progress made therein in both instrumentation and data‐analytical tools contributes to the general advances of portable NIR spectroscopy.

#### 3.1.4 Soil

Analysis of soil has prime importance for environmental research and agriculture. All conventional methods used for this purpose previously, in practice, required time‐consuming acquisition and transportation of contaminated soil samples to the laboratory. Therefore, portable NIR spectrometers have rapidly attracted much attention for this application. The analytical feasibility of miniaturized NIR spectrometers for such tasks has been exhaustively studied in the literature.[^83^, ^84^] For example, O'Brien et al. developed a method for analyzing hydrocarbon contamination in soil using a MicroNIR spectrometer.^[83]^ Highly accurate and reliable quantitative analysis of hydrocarbon contaminant in soil was possible, in spite of the heterogeneity of this type of sample. Soon after, a similar study by Altinpinar et al. demonstrated that microPHAZIR, a MEMS‐Hadamard spectrometer, was able to rapidly analyze hydrocarbon contaminants (gasoline, diesel, and oil) in soil, while maintaining standard errors of cross validation between 0.3 and 0.5 % (w/w), depending on the type of soil.^[84]^ These early studies demonstrated the high potential of miniaturized NIR spectrometers to be employed for on‐site, real‐time evaluation of contaminated soil for remediation applications. Keen interest in the use of NIR spectroscopy for analysis of soil quality and contamination can be deducted from a number of recently published articles (e.g., refs. [85, 86, 87]).

Importantly, soil characterization itself remains of keen interest in agriculture. The classification of soil types and the prediction of the physical, biological, and chemical properties of a soil and its quality for cultivation, typically required complex, resource‐intensive methods, often combining field observations and laboratory analysis.^[88]^ Therefore, the application of miniaturized NIR spectrometers for such a task offers decisive gains. A study by Lopo et al. demonstrated the feasibility of using a portable dispersive NIR spectrometer (Model NIR‐512, Ocean Optics) to classify vineyard soils.^[88]^ Samples of topologically similar origin were identified with 75 to 100 % accuracy, depending on the soil type. The performance of a portable instrument was compared with a benchtop one; interestingly, both delivered similarly good results. Tang et al. compared the performance of four different compact/transportable Vis/NIR instruments, including a NeoSpectra miniaturized NIR spectrometer, in the rapid analysis of soil towards predicting various properties: clay, sand, and total carbon (TC) content; cation‐exchange capacity (CEC); pH; and exchangeable Mg and Ca content.^[89]^ Despite a narrow operating spectral window, the NeoSpectra device performed admirably, with slightly lower accuracy only in predicting TC, sand, and clay contents.^[89]^

In another study, a miniaturized NIR spectrometer (NeoSpectra) was compared with a high‐performing compact benchtop instrument operating over the Vis/SW‐NIR/NIR spectral regions (350–2500 nm). The analysis aimed to determine the organic carbon (OC) and TC content in soil, which are important parameters of land suitability for crop cultivation. Although the benchtop instrument offered better performance, the NeoSpectra device yielded statistically acceptable predictions, which were concluded to be satisfactory for the intended purpose. However, in this case, the spectral analysis was preceded by in‐laboratory sample preparation procedures (drying, grinding, and sieving). Further studies validating miniaturized NIR spectroscopy as a fully in situ method are necessary. Similar comments may be made about the recent analyses of soil carbon and nitrogen contents by using the MicroNIR spectrometer.^[90]^

#### 3.1.5 Forensics and security

Portable NIR spectroscopy offers significant potential for detecting illegal drugs and euphoriants (see Section 3.1.1). Alongside the successes reported in a laboratory setting, studies on establishing a method directly applicable on‐site are currently being performed.[^91^, ^92^, ^93^] Recent feasibility studies presenting the capacity of portable NIR spectrometers to perform accurate on‐site determination of heroin^[92]^ and cocaine^[93]^ should be noted. In two other recent investigations, a MicroNIR spectrometer was successfully used to detect amphetamine^[94]^ and cocaine^[95]^ in human oral liquids. The performance of existing instrumentation and data‐analytical tools seem sufficient for such tasks and may be expected to be further refined with advancements in technology. However, further systematic investigations are necessary to ascertain the reliability of the analysis in real‐life applications. This seems an indispensable step into adopting NIR spectroscopy into existing security protocols.

Recently, it was demonstrated that portable NIR spectroscopy could successfully be used to authenticate banknotes.^[96]^ A case study showed 100 % accuracy in discriminating between authentic and counterfeit banknotes, based on a PLS‐DA model developed for NIR spectra measured with a MicroNIR spectrometer.

Interestingly, it was shown recently that miniaturized NIR spectroscopy was capable of analyzing blood stains,[^97^, ^98^] and that the sensitivity of the method did not seem to present a major issue. This shows the potential for NIR spectroscopy to compete with IR or Raman techniques, which are already in use for such purposes.[^99^, ^100^, ^101^, ^102^]

NIR spectroscopy has been demonstrated to be suitable for the detection of various explosive materials.^[103]^ An on‐site NIR analytical approach based on a microPHAZIR spectrometer was successfully developed and used in analyzing the chemical composition of explosives.^[104]^ Accurate determination of the oxidizer (RDX), stabilizer (Methylene di‐tertiary butyl phenol), and plasticizer (isodecyl pelargonate (IDP)) contents is essential to maintain the condition and stability of military warheads that use plasticized explosives. The application of a portable NIR spectrometer decreases safety hazards and largely improves the cost‐effectiveness of safety protocols. A study indicates that a similar method based on a NIR mini‐spectrometer could be used to control the condition of solid propellant in missiles.^[104]^ More recently, a fully on‐site screening method for the detection of explosives on human hands was developed on the basis of a MicroNIR spectrometer.^[105]^ It was demonstrated to be feasible to detect and classify, by means of PLS‐DA, trace amounts of explosives (<10 μg) in a complex matrix of the human skin surface. This accomplishment is an essential contribution along the path of establishing miniaturized NIR technology as a tool for detecting trace explosives.

NIR spectrometers demonstrated their usefulness in various successful applications in forensics and security. The particular potential of performing different types of analyses with a single, compact NIR instrument seems to be particularly promising for practical use in this field.

#### 3.1.6 Wood analysis

NIR spectroscopy has been established as a powerful analytical technique for the assessment of wood properties and quality.[^106^, ^107^, ^108^, ^109^] However, its full potential in this field was uncovered after the appearance of portable spectrometers. This essential breakthrough enabled effective NIR on‐site analysis in forestry.[^110^, ^111^, ^112^, ^113^] Similar to the previously overviewed fields of application, attention has been given to systematic evaluation studies on the applicability and performance, in a qualitative and quantitative sense, of portable NIR spectrometers in relation to laboratory equipment. Recently published examples include the evaluation of miniaturized instrumentation well established in other areas, for example, the popular MicroNIR spectrometer. The performance and applicability of this instrument, in comparison with a benchtop spectrometer, were determined for discriminating between wood sawdust samples collected from two *Eucalyptus* species, *E. pellita* and *E. benthamii*.^[114]^ PCA, LDA, and PLS‐DA chemometric tools were used to predict the origin of unknown samples; only the last two yielded the intended results. While the MicroNIR instrument was concluded to be inferior in this application to that of the benchtop instrument, it still offered a performance level that was satisfactory for the intended use. Interestingly, the authors concluded that the observed difference in performance was likely to result from a higher scatter profile observed in spectra measured by the miniaturized spectrometer. This factor led to a lesser robustness and a more critical importance of performing spectral acquisition with care if using a miniaturized spectrometer.^[114]^

Recent literature in this field notes that a particular requirement for ruggedness and capacity to operate in a harsh environment appears for the portable instrument.^[115]^ This creates the need for the development of specialized, yet cost‐effective, spectrometers by utilizing modules existing on the market. A prototype instrument developed by Sandak et al. in 2020 used a rugged metal case, while maintaining low‐power consumption, high cost‐effectiveness, and performance levels suitable for detecting wood defects.^[115]^ Importantly, the specialized instrument implements adjustable focusing optics to improve its applicability to collecting NIR spectra of chained wood logs that feature profound surface roughness and irregularities. Such surfaces decrease the quality of spectra measured by standard spectrometers, and the surface itself is not suitable for on‐site preparation.

#### 3.1.7 Polymers and textiles

Polymer analysis by portable NIR spectrometers may find critical importance in various applications. Recent literature indicates that attention is being given to applying handheld NIR spectrometers for the analysis of various polymers. For example, a study by Yan and Siesler comprehensively evaluated four different miniaturized NIR spectrometers (NeoSpectra, NIRONE Sensor, NIRscan Nano, and MicroNIR) in the qualitative and quantitative analyses of several polymers of major importance for industry and the environment: polyethylene, polypropylene, polyethylene terephthalate (PET), polyvinyl chloride, and polystyrene.^[116]^ Samples in different morphologies (pellets, films, plates, fibers, and powders) were used. The study provided a detailed analysis of the classification performance of these four spectrometers. In conclusion, however, all evaluated instruments successfully identified test samples of all considered polymers.^[116]^ A noteworthy potential for the particularly important adoption of miniaturized spectrometers stems from environmental pollution and growing public concerns about the omnipresence of microplastics in the environment. NIR spectroscopy is a promising tool for monitoring the threat arising from these pollutants.^[117]^

Portable NIR spectrometers were also assessed for their performance and applicability to textile analysis.[^118^, ^119^] Rodgers et al. demonstrated that three evaluated portable NIR spectrometers offered good performances in analyzing cotton fiber micronaire.^[118]^ The key quality parameters of this textile, maturity and fineness, were successfully determined by using miniaturized NIR instruments. Conventional methods of controlling the properties of micronaire require resource‐intensive in‐lab procedures with tightly controlled environmental conditions. Therefore, the ability to rapidly assess fiber micronaire, maturity, and fineness on‐site under ambient conditions by portable NIR spectroscopy is a major step forward in textile analysis. Yan and Siesler emphasized the potential of using handheld NIR spectrometers for textile authentication.^[119]^ They provided evidence that miniaturized instruments performed adequately in identifying most common textile types (poly(acrylonitrile)/acrylic, wool/cashmere, cotton, elastane, Kevlar, Nomex, PA6/PA66, PET, and silk).

#### 3.1.8 Fuel

The potential of miniaturized NIR spectrometers for fuel analysis was demonstrated, for example, by Lutz et al.^[120]^ The developed method was able to successfully predict the content of bioethanol in gasoline. Importantly, this study highlighted the importance of maintaining stable conditions for measurement in the case of portable spectrometers. First, a custom transflectance sampling cell was designed for the highly reproducible measurement of liquid samples by using a fused silica cuvette with a fixed optical pathlength. The sampling cell was equipped with a spherical gold mirror, for directly focusing the incident beam onto the detector. This solution was deemed mandatory for the quantitative analysis of liquid samples with the miniaturized spectrometer being operated by hand. Second, it was determined that the temperature drift of the miniaturized spectrometer must be corrected. This was accomplished through a purpose‐built thermoelectric cooling system for the MicroNIR spectrometer, largely improving its thermal stability, regardless of the heat built up in the device itself over the measurement time or external conditions. These custom solutions were concluded to be advantageous for increasing the accuracy of the collected spectral data sets and for highly reproducible quantification of ethanol in fuel by the MicroNIR spectrometer.^[120]^

**Figure 4:**
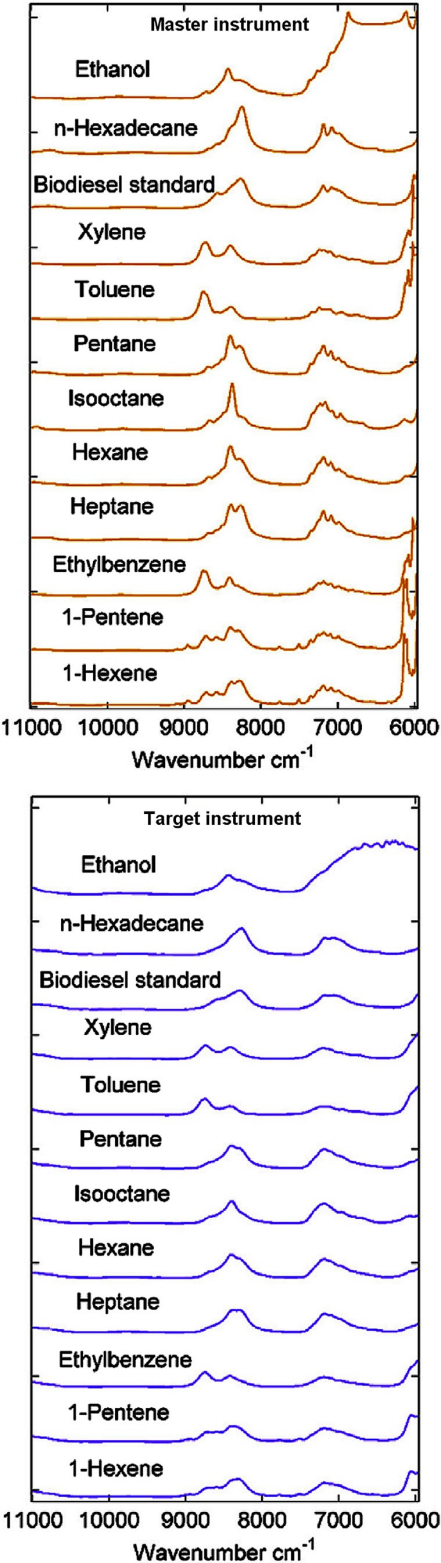
Raw spectra of 12 pure solvents measured on the master (benchtop) and target (miniaturized MicroNIR) instrument. Adopted with permission from ref. [121]. Copyright Elsevier, 2017.

The MicroNIR spectrometer, alongside a benchtop instrument, was also used by da Silva et al. for determining the quality parameters for gasoline and diesel/biodiesel blends.^[121]^ This study also aimed to develop an efficient calibration transfer method for use in the practical analysis of fuels, which involved an exhaustive number of chemicals (ethanol, pentane, 1‐pentene, hexane, 1‐hexene, heptane, toluene, isooctane, xylene, ethylbenzene, *n*‐hexadecane, and biodiesel B100 standard; Figure 4) that served as “virtual standards.” By proposing a virtual standards approach, there was no need for the measurement of standard samples and transfer between distant laboratories was achieved; this brings a considerable advantage in terms of security regulations in the fuel industry. A more efficient and less‐resource‐intensive operation of a miniaturized spectrometer on‐site may use this approach based on a calibration model developed under better controlled conditions and on a master (i.e., high‐performing benchtop) instrument.^[121]^

#### 3.1.9 Miscellaneous other issues

Miniaturized NIR spectroscopy shows potential as a tool for analyzing old photographs. The performance of a portable NIR spectrometer, in comparison with a benchtop ATR‐IR instrument, for the identification of historic photographic print materials was performed recently.^[122]^ NIR spectroscopy enabled a deeper sensing of the inner layer of photography. Interestingly, the authors concluded that the combined use of both techniques gave the unique capability to acquire more complete information of the sample. This observation opens up future potential for the use of combined sensors in a single instrument in similar applications, for which relevant information is distributed in‐depth in the sample of interest.

In medical diagnosis, specialized sensors are preferred, rather than commercially available sensors intended for general use. However, progress in technology and data‐analytical approaches brings direct benefit and contributes to the advances made in this specialized field as well. Examples of recent research activity in this field can be provided, for example, refs. [123], [124].

Considerable attention is given in the literature to the concept of calibration transfer.^[51]^ The ability to use standardized calibrations, preferably performed in the laboratory with the use of a benchtop spectrometer, and further used on‐site, possibly with different portable instruments brings decisive practical gains in several fields of application. Recent research activity in this area includes studies by Hoffmann et al.^[125]^ and da Silva et al.^[121]^

## 4 Summary and Future Prospects

The advent of miniaturized spectrometers initiated a significant turning point in the evolution of practical applications of NIR spectroscopy. This breakthrough led to the appearance of this analytical technique in a variety of new, previously unattainable, scenarios. The capacity for rapid, nonintrusive on‐site analysis is a decisive improvement over conventional methods used in numerous fields. For this reason, one has observed the rapidly increasing utilization of miniaturized NIR spectrometers over the last few years. Recent literature delivers rich evidence of the potential of portable NIR spectroscopy and its importance for modern analytical chemistry. In the near future, extremely compact and cost‐effective spectrometers may lead to NIR spectroscopy crossing the barrier between professional and consumer use.

In general, two directions of advancements in miniaturized NIR spectroscopy may currently be observed. The most propitious development trend in the technology is being achieved for wavelength selectors. These elements are becoming more compact and performing better, at the same time. Good examples may be provided by the new generation of MEMS Michelson interferometers, for example, those introduced by Hamamatsu^[29]^ or Hefei SouthNest Technology,^[28]^ in which the limitations in optical throughput, often faced by earlier generations of interferometer‐based instruments, are markedly improved. These instruments should offer better S/N and a notably wider operating spectral region. On the other hand, recent designs utilizing Fabry–Pérot interferometers have achieved an impressive level of miniaturization and utility factor, for example, the mechanically rugged and hermetically sealed sensor introduced by Hamamatsu with a weight of less than 1 g.^[31]^ Progress in technology significantly improves the application potential of the new generation of instruments. Their use in more challenging measurement conditions becomes feasible, while the improved performance (e.g., coverage of almost the entire NIR spectral region, better quality of the spectra) makes them suitable for a wider range of analyses. Another pursued direction of advancement can be seen in attempts to make spectrometers more suitable for operation by nonprofessional personnel and regular consumers, with control software preconfigured for typical analyses.

Nevertheless, the high level of miniaturization comes with advantages and disadvantages compared with the analytical performance of compact instruments. Furthermore, unlike a mature FTNIR benchtop spectrometer, these novel designs implement various technologies and differ in operational characteristics. Therefore, their performance profiles are distinct in device‐specific ways. The systematic evaluation of the applicability and analytical performance of various miniaturized NIR spectrometers in different scenarios is presently an extremely active area of research. The picture obtained from the literature indicates that the performance of different miniaturized NIR spectrometers depends on a number of factors, for example, chemical composition and physical properties of the sample. Often, the performance of miniaturized NIR spectrometers is concluded to be adequate, while the practical gains from using a portable analyzer are fundamental. Furthermore, the miniaturization versus performance factor continuously improves with new spectrometers pushing the frontier of this technology; for example, mini‐FTNIR spectrometers that operate over the entire NIR spectral region were announced in 2020. Reports in the literature suggest that often spectra‐analytical procedures based on miniaturized NIR spectrometers require more supervision than is the case for in‐lab NIR spectroscopy if the analytical performance is prioritized. It may be envisioned that future efforts will also be aimed at overcoming this issue. Current research efforts are essential to better direct the future development of miniaturized NIR spectroscopy.

## Conflict of interest

The authors declare no conflict of interest.

## Biographical Information

*Krzysztof B. Beć obtained his doctorate (2014) in physical and theoretical chemistry from the University of Wroclaw. His research focused on thin‐film IR spectroscopy and computational methods. He worked with Professor Yukihiro Ozaki as Postdoctoral Fellow and Research Assistant Professor at Kwansei Gakuin University, Japan; he focused on advancing near‐IR spectroscopy and contributed into development of ATR‐FUV‐DUV spectroscopy and its applications. He continues his work in the field of NIR spectroscopy as FWF Lise Meitner Senior Fellow in the group of Professor Christian W. Huck at the University of Innsbruck, Austria*.

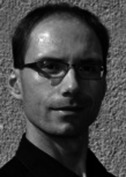

## Biographical Information

*Justyna Grabska obtained her doctorate (2015) in physical and theoretical chemistry from the University of Wroclaw, Poland. Her thesis focused on advancing vibrational spectroscopy in physical chemistry. She expanded her research field into analytical spectroscopy after joining the group of Professor Christian W. Huck as Postdoctoral Fellow at the University of Innsbruck, Austria. She was Postdoctoral Researcher in the group of Professor Yukihiro Ozaki, where she focused on computational near‐IR spectroscopy and its applications to biomolecules. She is currently continuing her work in the group of Professor Christian W. Huck, with a focus on analytical spectroscopy*.

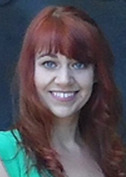

## Biographical Information

*Christian W. Huck obtained his doctorate in chemistry in 1998 from the University in Innsbruck, Austria, where he continued to work as Assistant Professor until his habilitation in 2006. In 2013, he became Full Professor at the University of Stuttgart, Germany, and in 2015, moved back to the University of Innsbruck, where he is currently Vice‐Head of the Institute of Analytical Chemistry and Radiochemistry and Head of the spectroscopy unit. From 2014 until 2017, he was Visiting Professor at the Kwansei‐Gakuin University in Sanda, Japan, in the laboratory of Professor Yukihiro Ozaki. He has published more than 300 peer‐reviewed manuscripts, resulting in an* h *index of 50 based on 8570 citations (Google Scholar). In additional to several awards, he was also the recepient of the 2018 Tomas Hirschfeld Award*.